# Quality of Chronic Anticoagulation Control in Patients with Intracranial Haemorrhage due to Vitamin K Antagonists

**DOI:** 10.1155/2018/5613103

**Published:** 2018-08-02

**Authors:** Carlos Estevez-Fraga, Maria Molina-Sanchez, Rodrigo Alvarez-Velasco, Pablo Agüero-Rabes, Leticia Crespo-Araico, Elena Viedma-Guiard, Antonio Cruz-Culebras, Consuelo Matute, Rocio Vera, Alicia De Felipe-Mimbrera, Jaime Masjuan Vallejo

**Affiliations:** ^1^Neurology Department, Ramon y Cajal University Hospital, Madrid, Spain; ^2^Neurology Department, Rey Juan Carlos University Hospital, Mostoles, Spain; ^3^Neurology Department, Fundacion Jimenez Diaz, Madrid, Spain; ^4^Neurology Department, Principe de Asturias University Hospital, Alcala de Henares, Spain; ^5^Department of Neurology and Stroke Center, Bichat Hospital, Paris, France

## Abstract

**Introduction:**

Patients treated with vitamin K antagonists (VKA) are at increased risk of intracranial haemorrhage (ICH). The purpose of our study was to determine the quality of previous anticoagulation control in patients with VKA-associated ICH.

**Materials and Methods:**

We prospectively assessed every consecutive patient admitted to our stroke unit with VKA-associated ICH between 2013 and 2016. Demographic, clinical, and radiological variables, as well as consecutive international normalized ratios (INR) during 7 previous months, were extracted. Time in therapeutic range (TTR), time over range (TOR), time below range (TBR), and percentage of INR within range (PINRR) were calculated.

**Results and Discussion:**

The study population comprised 53 patients. Mean age was 79 years; 42% were women. Forty-eight patients had atrial fibrillation (AF) and 5 mechanical prosthetic valves. Therapeutic or infratherapeutic INR on arrival was detected in 64.4% of patients (95% CI 2.7 to 3.2). TTR was 67.8% (95% CI: 60.2 to 75.6 %) and PINRR was 75% (95% CI: 49.9-100). TOR was 17.2% (95% CI: 10.4 to 23.9% ) and TBR was 17% (95% CI: 10.6 to 23.9%).

**Conclusion:**

VKA-associated ICH happens usually in the context of good chronic anticoagulation control. Newer risk assessment methods are required.

## 1. Introduction

Oral anticoagulant (OAC) medication is the mainstay of prophylaxis for embolism. It reduces the risk of cardioembolic stroke by 66% in patients with atrial fibrillation (AF) [1] and by 75% in those with mechanical heart valve prostheses [2]. However, it increases the risk of intracranial haemorrhage (ICH), which is the most feared and lethal complication of this therapy [3, 4].

Treatment with OACs accounts for approximately 20% of all cases of ICH, most of which are associated with the vitamin K antagonists (VKAs), acenocoumarol and warfarin [5]. ICH affects 0.3-1.5% of patients taking VKAs [5, 6] and is more severe than non–VKA-associated ICH [5]

Advancing age and newer AF detection methods [7, 8] have led to an increase in the prevalence of AF [9–11] and OAC use. Findings on anticoagulant medication and risk of complications in patients taking VKAs are mixed. While most publications found that a lower time in the therapeutic range (TTR) was associated with an elevated risk of both haemorrhage and ischemia [12–16], few studies have specifically addressed ICH. Moreover, most studies did not carefully examine other major anticoagulation parameters such as the time over range (TOR), time below range (TBR), and percentage of international normalized ratios (INR) in the therapeutic range (PINRR).

Assessment of anticoagulation on admission using the INR has been a matter of debate. INR on admission is usually within range and does not correlate with prognosis or with ICH volume [17]. Hence, reversal of anticoagulation with vitamin K, fresh frozen plasma, or prothrombin complex does not appear to affect outcomes although evidence comes from observational studies and not from randomized controlled trials [18, 19].

In addition, publications on the precise clinical characteristics of OAC-induced ICH and its relationship with long-term management of OAC are scarce [20]. With the arrival of safer direct oral anticoagulants (DOAC) and restrictive government regulations on switching from VKAs in some countries, which are based mainly on long-term control of OAC medication, the association between ICH and VKAs seems more crucial than ever.

Our objective was to determine the quality and characteristics of OAC managed with VKAs in patients admitted to our institution with ICH. Secondary endpoints included prognosis of VKA-ICH and treatment used to prevent systemic embolism after ICH.

## 2. Materials and Methods

We conducted a prospective, single-centre observational study of every consecutive patient admitted to the Stroke Unit of Ramon y Cajal University Hospital, Madrid, Spain, because of VKA-related intraparenchymal ICH between July 2013 and December 2016. We included patients anticoagulated owing to AF or mechanical heart valves. We excluded patients with subdural haematomas, subarachnoid haemorrhages, and haemorrhagic transformation of ischaemic stroke. Our hospital is a comprehensive stroke centre having a catchment population of 1,000,000 people that forms part of the Madrid Stroke Network (MSS). MSS is composed of nine hospitals provided with stroke units and 17 community hospitals working within a coordinated system to guarantee specialized care for all stroke patients in the region of Madrid, with a population of around 6.3 million inhabitants.

On admission, we assessed previous vascular risk factors, conditions requiring treatment with anticoagulant medication, and previous thromboembolic and haemorrhagic risk scores (CHA_2_DS_2_-VASc and HASBLED) [21, 22]. We also examined additional previous treatment with antiplatelet agents and presence or absence of leukoaraiosis on cranial computed tomography (CT) scans on admission.

We prospectively recorded vital signs, National Institute of Health Stroke Scale (NIHSS) score on admission, seizures, and the modified Rankin scale (mRS) score before the stroke and 3 months after the stroke. We also recorded clinical worsening during admission, repetition of CT scans, haematoma expansion, performance of magnetic resonance imaging (MRI), length of stay, location of the haemorrhage, ICH volume calculated using the ABC/2 formula [23], and the presence/absence of underlying structural lesions or intraventricular clot. We gathered data on treatment of ICH (medical with reversal agents and/or surgery). Anticoagulant treatment at discharge and 3 months after ICH were also reviewed.

We collected the INR during the previous 7 months and calculated the TTR, TOR, TBR, and PINRR based on the Rosendaal method [24]. We excluded patients where INR data were incomplete or not available. We also evaluated INR on admission and other coagulation parameters. Therapeutic ranges were considered to be between 2 and 3 for AF and between 2.5 and 3.5 for mechanical prosthetic valves.

Data were analysed using Microsoft Excel 2013® and Stata SE 12.0®.

## 3. Results

### 3.1. Study Population (Table 1)

We included a total of 53 consecutive patients receiving VKAs. Forty-eight were anticoagulated owing to AF, and 5 had mechanical prosthetic heart valves. Mean age on admission was 79 years (interquartile range [IQR] 76-84), and 26 (48%) patients were women. 91% were independent (mRS 0-3) before ICH. Seven patients had had a previous stroke, which had been haemorrhagic in 4 cases. The median CHA_2_DS_2_-VASc score was 4 (IQR, 3-5). The median HASBLED was 2.5 (IQR, 2-3). Three patients were taking antiplatelet agents and 19 statins.

### 3.2. Characteristics of ICH (Tables 2 and 3)

#### 3.2.1. Clinical

The median NIHSS on admission was 13 (IQR, 4-20). After 3 months, only 13 patients (24%) remained independent (mRS 0-3) and 34 (64%) had died. The mean length of hospital stay was 7 days. Clinical worsening during hospitalization was recorded in 33 patients (61%). Imaging was repeated in 26 (50%) and disclosed haematoma expansion in 59% of those who underwent further CT or MRI.

#### 3.2.2. Radiological

Mean ICH volume was 24 cm^3^. Only 2 patients had underlying structural lesions (cavernomas). The location was heterogeneous: basal ganglia, 38 %; lobar, 29%; intraparenchymal extensive, 17%; primary intraventricular, 2%; posterior fossa, 15%. Intraventricular extension was present in 38.4%.

#### 3.2.3. Treatment

Care was withdrawn on admission in 11 patients, all of whom died. Further 5 patients did not receive reversal of anticoagulation or surgery. 26 patients were treated with vitamin K and prothrombin and 6 patients were treated with vitamin K alone. Four patients were treated surgically (2 with decompressive craniectomy and 2 with ventricular drain placement) and we do not have data about anticoagulation reversal therapy. In one patient data were not available.

Upon discharge, 3 patients (12.5%) received antiplatelet medication, 2 (8.3%) VKAs, 2 (8.3%) DOAC, and 5 (20.8%) prophylactic low-molecular-weight heparin. Ten patients (41.6%) did not receive any antithrombotic treatment immediately after discharge.

After 3 months, only 2 of the surviving patients (9.1%) continued to receive antiplatelet agents, further 2 (9.1%) received acenocoumarol because both had a mechanical heart valve, 13 (59.1%) received DOAC, and 3 (13.6%) underwent left atrial appendage closure. Overall, in 15 out of 22 patients, treatment was changed 3 months after discharge, and there were no patients without treatment at this point. Neither thrombotic nor haemorrhagic events were detected during follow-up.

#### 3.2.4. Prognostic Factors (Table 4)

The only statistically significant differences between patients who died and survivors were found for ICH volume (33.9 versus 12.5 cm^3^, p<0.0002), INR on admission (3.3 versus 2.5, p<0.01), and NIHSS score on admission (25 versus 6, p<0.0001).

### 3.3. Anticoagulation

#### 3.3.1. INR on Admission

Median INR on admission was 2.71 (IQR, 2.4-3.5) for patients with AF and 4.01 (IQR, 3.7-4.6) in patients with mechanical heart valves. On admission, 29 (54.7%) patients were within therapeutic range and 20 (37.7 %) had supratherapeutic INR and 4 (7.5 %) had infratherapeutic INR. We did not find a statistically significant correlation between INR on admission and location of the ICH.

#### 3.3.2. Long-Term Anticoagulation

Mean TTR during the previous 7 months calculated using the Rosendaal method was 67% (95% CI: 60.2 to 75.6 %), and the PINRR was 75% (95% CI: 49.9-100). TOR was 17.2 % (95% CI: 10.4 to 23.9% ) and TBR was 17% (95% CI: 10.6 to 23.9%).

## 4. Discussion

Our series confirms the gloomy prognosis of VKA-ICH [9, 25, 26], with mortality rates over 50% after 3 months and high rates of disability. These results are especially striking when previous mRS are taken into account, with almost 9 out of every 10 patients being independent before ICH.

Mortality was significantly associated (p<0.05) with only 3 variables: higher INR on admission, higher NIHSS scores, and larger ICH volumes on admission. The INR level on admission has not been consistently associated with prognosis of ICH [19, 27] probably because of longer times from onset of symptoms to CT during previous years, when stroke symptoms were underrecognized by the general population.

As in previous series, location of the ICH was also heterogeneous and similar to non–VKA-related ICH [25]. The latter finding contrasts with the generally held belief that lobar ICH is more common in patients treated with OAC than hypertensive haemorrhages. No differences in location were observed when patients were stratified according to INR levels on admission.

We also found that even though INR is a prognostic factor, most patients do not present with supratherapeutic INR on admission [10, 28]. In fact, in our sample, supratherapeutic INR were seen in 37.7 % of patients, whereas 7.5% presented with infratherapeutic levels of anticoagulation. These data should be taken with caution due to the small numbers but could imply an INR-dependent risk of haemorrhage that is increased, even when therapeutic levels have been achieved, possibly owing to various etiologies that predispose to ICH. The increased likelihood of ICH was not due to the fact that patients were undergoing adjustment of OAC, as every patient had been on treatment for at least 7 months.

We did not find a relationship between TOR and TBR in ICH, as suggested by previous studies [12], since TOR and TBR were very similar in the present study.

We examined trends in resumption of anticoagulation treatment after ICH and found that only 10% of surviving patients did not receive anticoagulant treatment after discharge. Of the remaining patients, none of those who underwent left atrial appendage (LAA) closure or those who were anticoagulated experienced thrombotic or haemorrhagic events during follow-up. This finding differs from those of Poli et al. [29] and might be related to the small size of our sample, short follow-up, and use of DOAC instead of VKAs in our series. As LAA closure was still being implemented while data were being collected, it is highly likely that more patients will undergo this procedure in the future. However, DOAC and LAA closure [30, 31] have both proven to be at least as effective as VKAs for preventing stroke without increasing the risk of haemorrhage.

In many developed countries, the arrival of DOAC has triggered strict administrative regulations regarding switching from VKAs, which requires deficient TTR during the previous months. Nevertheless, we found that anticoagulation was generally well controlled in patients with ICH: the mean TRT was 67%, which is above Spanish government requirements for switching treatment (65%) [32].

Our study has several limitations. First, it is a single-centre study that might be influenced by the characteristics of the study population, by local general practitioners' expertise in controlling anticoagulation, and by our own clinical practice. However, a good global TTR has previously been reported in our country [33]. Another drawback is that although patients were included prospectively, we retrospectively collected their previous INR. In addition, we also lacked a control group, which would have allowed us a direct comparison of anticoagulation control in patients with and without ICH.

## 5. Conclusion

In summary, we found that VKA-related ICH is a devastating disorder that tends to affect patients with well-managed long-term anticoagulation medication, as measured by TTR and other indexes. VKA-related ICH can be partially avoided with easier and earlier access to newer and safer treatments such as DOAs.

## Figures and Tables

**Table 1:** Baseline characteristics.

**BASELINE CHARACTERISTICS (N=53)**	
Age, median (range) years	79 (56-93)

Sex (female)	26 (42%)

**Previous mRS **N (%)	
(i) 0-3	41 (91%)
(ii) 4-5	4 (9%)

Arterial hypertension N (%)	4 (9%)

Diabetes mellitus N (%)	13 (25%)

Dyslipidemia N (%)	23 (43%)

**Previous stroke:**	
(i) TIA N (%)	7 (13.21%)
(ii) Ischaemic N (%)	2 (5%)
(iii) Haemorrhagic N (%)	3 (6.5%)
(iv) Both N (%)	2 (5%)

HASBLED median (p25-p75)	2.5 (2-3)

CHAD_2_S_2_-VASc median (p25-p75)	4 (3-5)

**Condition requiring anticoagulation:**	
(i) Atrial fibrillation N (%)	48 (90.6%)
(ii) Mechanical heart valves N (%)	5 (9.4%)

**Concomitant medications:**	
(i) Antiplatelet agents N (%)	3 (6.5%)
(ii) Statins N (%)	19 (43.2%)

Leukoaraiosis N (%)	24 (47%)

TIA: transient ischaemic attack. VKA: vitamin K antagonist.

**Table 2:** Characteristics of ICH.

**CLINICAL AND RADIOLOGICAL CHARACTERISTICS OF ICH**	
**Length of stay**, (days, range)	7 (1-38)

**SBP on admission **(mmHg, range)	160 (110-242)

**DBP on admission **(mmHg, range)	81.5 (40-146)

**HR on admission **(bpm, range)	79 (41-150)

**Glycemia on admission **(mg/dl, range)	137 (76-280)

**ICH volume** (cm)	23.7 (mean=15)

**Baseline NIHSS **	13 (0-42)

**NIHSS at discharge**	16 (0-42)

**NIHSS at discharge **(survivors)	2 (0-16)

**Structural lesion**	
(i) None	51 (96%)
(ii) Cavernoma N (%)	2 (4%)

**In-hospital mortality** N (%)	27 (51%)

**3-month mortality ** N (%)	29 (64.4%)

**Intraventricular extension ** N (%)	20 (38.4%)

**Seizures ** N (%)	2 (4%)

**Clinical worsening ** N (%)	27 (61.4%)

**Repetition of imaging studies ** N (%)	22 (50%)

**Haematoma expansion ** N (%)	13 (59%)

**Withdrawal of care ** N (%)	11 (25%)

**mRS after 3 months **N (%)	
(i) 0-3	13 (29%)
(ii) 4-6	40 (71%)

**Haemorrhage location **N (%)	
(i) Deep	20 (38%)
(ii) Lobar	15 (29%)
(iii) Intraparenchymal extensive	9 (17%)
(iv) Intraventricular	1 (2%)
(v) Brainstem	6 (11.5%)
(vi) Cerebellum	2 (4%)

ICH: intracranial haemorrhage. SBP: systolic blood pressure. DBP: diastolic blood pressure. HR: heart rate. NIHSS: National Institutes of Health Stroke Scale. mRS: Modified Rankin Scale. Values are shown as median (range) unless otherwise specified.

**Table 3:** Treatment.

**TREATMENT**	
**Haemorrhage treatment **N (%)	
(i) None	16 (30.8%)
(ii) Vitamin K	6 (11.5%)
(iii) Prothrombin	0 (0%)
(iv) Prothrombin and vitamin K	26 (50%)
(v) Surgical	4 (7.7%)

**Treatment at discharge **N (%)	
(i) None	10 (41.6%)
(ii) Antiplatelet agents	3 (12.5%)
(iii) VKAs	2 (8.3%)
(iv) VKAs and antiplatelet agents	0 (0%)
(v) Dabigatran	2 (8.3%)
(vi) Rivaroxaban	0 (0%)
(vii) Apixaban	0 (0%)
(viii) NOAC and aspirin	0 (0%)
(ix) LAA closure	5 (20.8)
(x) Prophylactic heparin	

**Treatment at 3 months **N (%)	
(i) None	0 (0%)
(ii) Antiplatelet agents	2 (9.1%)
(iii) VKAs	2 (9.1%)
(iv) VKAs and antiplatelet agents	0 (0%)
(v) Dabigatran	4 (18.2%)
(vi) Rivaroxaban	7 (31.8 %)
(vii) Apixaban	2 (9.1%)
(viii) NOAC and aspirin	0 (0%)
(ix) LAA closure	3 (13.6%)
(x) Prophylactic heparin	1 (4.5%)

**Treatment changed 3 months after discharge.**	15/22

VKA: Vitamin K antagonist. NOAC: novel oral anticoagulant. LAA: left atrial appendage.

**Table 4:** Differences between patients who died and survivors.

	**Patients who died (N=29)**	**Survivors (N=22)**	
**Age** mean (years)	78	80	n.s.

**SBP** mean (mmHg)	170	156	n.s.

**DBP** mean (mmHg)	89	81	n.s.

**HR** mean (bpm)	83	79	n.s.

**Glycemia** mean (mg/dl)	160	132	n.s.

**HASBLED** median	2.8	2.4	n.s.

**CHAD**_**2**_ **S**_**2**_ **-VASc** median	3.5	4.23	n.s.

**NIHSS** median	25	6.1	p<0.05

**Leukoaraiosis** %	39%	47%	n.s.

**Intraventricular extension** %	56%	33%	n.s.

**Haemorrhage location** N (%)			
(i) Deep	18.5%	41.6%	n.s.
(ii) Lobar	29.6%	45.8%	n.s.
(iii) Intraparenchymal extensive	14.8%	8.3%	n.s.
(iv) Intraventricular	33.3%	0	n.s.
(v) Brainstem	0 %	8.3%	n.s.
(vi) Cerebellum	3.7%	0 %	n.s.

**ICH volume** (cm^3^)	33.9	12.5	p<0.05

**INR on admission **(mean)	3.3	2.5	p<0.05

n.s.: not statistically significant differences. SBP: systolic blood pressure. DBP: diastolic blood pressure. HR: heart rate. VKA: vitamin K antagonist. NIHSS: National Institutes of Health Stroke Scale.

## Data Availability

The data used to support the findings of this study are available from the corresponding author upon request.
